# Ageism Healthcare: Implications for the Psychological Well-Being of Atlantic Canadian Healthcare Professionals

**DOI:** 10.1177/07334648241237099

**Published:** 2024-03-21

**Authors:** Madelyn Purchase, Éric R. Thériault, Brooke Collicutt

**Affiliations:** 1 School of Physiotherapy, 70338 Dalhousie University, Halifax, NS, Canada; 2 Department of Psychology, 55964 Cape Breton University, Sydney, NS, Canada; 3 Social Work Program, 55964 Cape Breton University, Sydney, NS, Canada

**Keywords:** ageism, caregiving, well-being

## Abstract

Ageism in healthcare is related to poor outcomes for older patients. The objective of this study was to evaluate the relationships between ageism and various aspects of the psychological well-being of healthcare professionals in Atlantic Canada. In 2023, an online survey of 294 healthcare professionals from various disciplines was conducted. This survey included items to measure expectations of aging, stress, burnout, emotional dissonance, and well-being. Results indicated that aging expectations were significantly related to burnout, perceived stress, well-being, and emotional dissonance. With the use of a path analysis, emotional dissonance partially mediated relationships between burnout and well-being with stress. However, aging expectations did not significantly predict emotional dissonance. Differences were found across professional groups on ageism. Conclusions support the need for increased awareness to the relationship between ageist attitudes and professionals’ well-being, as well as the need for education and interventions to reduce false expectations about the aging process.

**What this paper adds**

• A novel examination of the impact of ageism on healthcare professionals.
• An insight into the attitudes of various healthcare professions toward aging.
• Inclusion of emotional dissonance in the examination of ageism and burnout.

**Applications of the study findings**

• Might be used to develop professional training and education initiatives.
• Can be applied to implement workplace interventions to deal with ageism, stress, and burnout.
• Findings can be used to advocate for policy changes and organizational initiatives within healthcare institutions.

## Introduction

Ageism and ageist attitudes within healthcare systems are problematic, especially since they influence the care received by older adults (Ben-Harush et al., 2017; Schroyen et al., 2016). Ageism—the stereotyping, prejudice, and discrimination toward older adults (Butler, 1969; Nelson, 2016)—is especially prevalent among healthcare professionals (Ben-Harush et al., 2017) and may be reflected in negative beliefs and behaviors toward the aging population (Schroyen et al., 2016). Healthcare professionals may be at a higher likelihood of holding, or forming, ageist attitudes because of their close contact with dependent, ill, or dying individuals (Schroyen et al., 2016). Certain expectations of aging such as higher rates of depression, worry, body aches, and decreased energy levels are more accepted by primary care clinicians, compared to non-healthcare professionals. Similarly, social workers, physicians, and nurses were all more likely to associate difficulties, such as being a demanding client, with older rather than with younger individuals (Ben-Harush et al., 2017). The ageist attitudes of healthcare professionals may differ based on their professions; however, the literature is limited (e.g., Giles et al., 2002; Schroyen et al., 2016). It was also found that older and more experienced individuals tend to have higher levels of ageism (Punchik et al., 2021). Understanding these influences may help in the delivery of targeted interventions.

**Figure 1.**
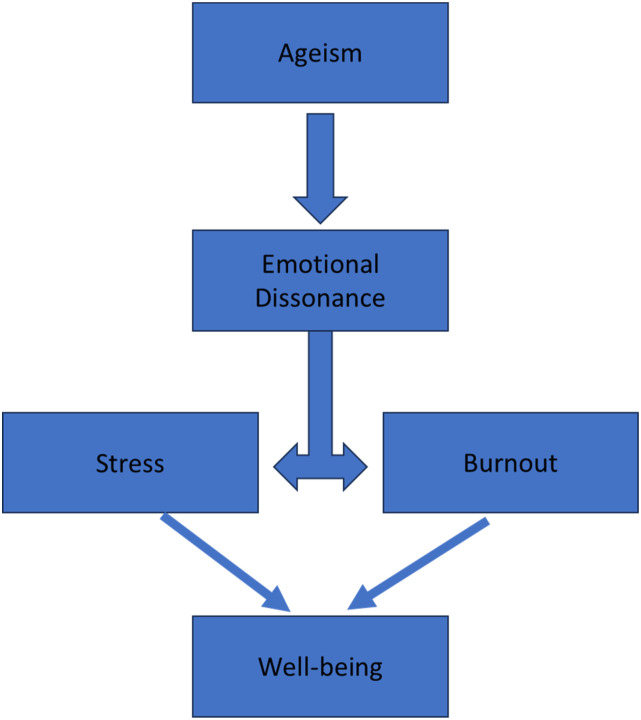
Conceptual description of the relationship between concepts.

Although ageism can put the health and safety of older adults at risk, it has also been found to pose risks to the psychological well-being of the professional. That is, holding ageist attitudes toward older individuals has been found to contribute to the development of burnout, see Figure 1 (e.g., Hakim, 2021).

Research on this relationship is very limited and was first reported by Iecovich and Avivi (2017), in nurses, where those with poorer attitudes toward older adults experienced a greater sense of burnout. However, the nature of the relationship between burnout and ageism is more complicated when examining the specific dimensions of burnout. Burnout is characterized by three dimensions: emotional exhaustion, depersonalization or cynicism, and reduced personal accomplishment or self-efficacy (Andela et al., 2015). The most researched component of burnout is emotional exhaustion (Maslach et al., 2001). In reaction to emotional exhaustion and demands, individuals may begin to seek greater distance from or dehumanize clients, which mark the depersonalization dimension of burnout (Maslach & Jackson, 1981). Depersonalization manifests itself as insensitive, insincere, and overall negative attitudes held by professionals, particularly toward their clients (Maslach et al., 2001; Maslach & Jackson, 1981).

In one study, it was revealed that nurses with poorer perceptions of older individuals were no more emotionally exhausted than their peers (Iecovich & Avivi, 2017). However, they were more likely to have a worsening sense of personal accomplishment and greater levels of depersonalization (Iecovich & Avivi, 2017). In a separate study, emotional exhaustion shared no relationship with ageism (Pekçetin, 2018). Recently, Hakim (2021) found that overall burnout, depersonalization, and nurses’ sense of accomplishment were all related to ageism. This study found that nurses with ageist attitudes were more emotionally exhausted than those who were low in the same attitudes (Hakim, 2021). Thus far, research on the ageism-burnout relationship has been limited to nurses.

Beyond its relationship with ageism, burnout is of particular concern to healthcare professionals, as they are more likely to experience burnout than non-healthcare professionals (Cañadas-De la Fuente et al., 2015). The rates of burnout in healthcare professionals are rising, and they are not showing any signs of slowing down (Cañadas-De la Fuente et al., 2015). In situations where an individual faces prolonged periods of stress or excessive demands in their workplace, the development of burnout is a common consequence (Bruschini et al., 2018). Burnout and stress have very similar effects on well-being. This may result in physical symptoms such as sleep disturbances and headaches, as well as poor mental health outcomes, including anxiety and depression (Moss et al., 2016). Given the close relationship between symptoms of stress and burnout (Mackintosh, 2007), these similarities are not shocking; however, they are concerning.

Similar to burnout, healthcare professionals are at an elevated risk of experiencing high levels of stress. Likely because they are exposed to a variety of excessive demands such as staff shortages, poor resource availability, low pay, the perception of working too many hours, shift work, role conflict, sleep deprivation, overtime hours, as well as an overload of clients or patients (e.g., Birhanu et al., 2018; Farrow et al., 2019). Although the frequency of particular stressors may differ across professions, the perception of a stressful work-life appears to be a common factor (Birhanu et al., 2018). In addition, there may be variations in what aspects of burnout that stress can predict. For instance, among medical secretaries, perceived stress was not related to personal accomplishment but was related to the other two dimensions of burnout (Devebakan, 2019). Different aspects of stress may also influence burnout differently, for example, excessive workloads contributed to both emotional exhaustion and depersonalization (Joaquim et al., 2018).

Considering the higher rates of stress and burnout in healthcare professionals, it is vital to better understand their role in the ageist beliefs of healthcare providers. Iecovich and Avivi (2017) proposed that one possible explanation for the relationship between ageism, stress, and burnout is emotional dissonance. This state occurs in situations where a professional is required, based on organizational or cultural expectations, to express emotions that differ from those that they feel (Zapf, 2002). For healthcare professionals, this may result from requirements to suppress emotions that are deemed inappropriate or potentially offensive while demonstrating the culturally preferred emotions (Andela et al., 2016). This discrepancy of emotions, relating to their ageist attitudes, may place healthcare workers in a state of emotional dissonance. This state itself is a significant source of stress and may contribute to the role of stress in the development of burnout in healthcare professionals (Andela et al., 2016). There is some evidence to suggest a relationship between emotional dissonance and the three components of burnout (Andela et al., 2015, 2016) as well as a relationship with stress (Karimi et al., 2014). However, the role of emotional dissonance on ageism has yet to be established.

The purpose of this study was to better understand stress, burnout, and ageism in healthcare professionals in Atlantic Canada and to understand the role of emotional dissonance in this relationship. To accomplish this, two research questions and two hypotheses were generated: RQ 1 - Does work experience and profession influence stress, burnout, and expectations regarding aging? RQ 2 - What is the relationship between stress and ageism? H1 - Poorer expectations regarding aging will be related to higher levels of overall burnout, emotional exhaustion, and depersonalization. H2 - this would also be related to lower levels of personal accomplishment and lower well-being scores via its influence on emotional dissonance.

### Methods

#### Participants and Procedure

In early 2023, an online questionnaire was completed by 294 healthcare professionals currently practicing and residing in one of the four Atlantic Canadian Provinces, consisting of Nova Scotia, New Brunswick, Prince Edward Island, and Newfoundland and Labrador. Of the 381 participants who were recruited through social media, email, and word-of-mouth, 87 of these surveys were discarded because they were incomplete and/or ineligible for the study. Participation in this study was voluntary (without compensation) and was not limited to specific healthcare settings and included a wide variety of healthcare professionals. A full breakdown of this can be found in Table 1. This study received REB approval from Cape Breton University.

**Table 1.** Demographic Characteristics.

Variable	Participant Characteristics
	% (*N*)
Gender	
Female	90.1% (265)
Male	9.9% (29)
Marital status	
Married	46.8% (137)
Not married	53.2% (164)
Children	
No children	44.2% (130)
Children	55.8% (164)
Mean = 1.2	
Work experience	
Less than 5 years	23.8% (70)
5–15 years	40.5 (119)
More than 15 years	35.7% (105)
Mean = 13.73	
Profession	
Registered nurse (RN)	33.0% (97)
Paramedic	13.6% (40)
Physiotherapist (PT)	12.6% (37)
Speech-language pathologist (SLP)	9.9% (29)
Licensed practical nurse (LPN)	8.2% (24)
Social worker (SW)	4.1% (12)
Audiologist	3.4% (10)
Pharmacist	3.1% (9)
Occupational therapist (OT)	3.1% (9)
Physician	1.7% (5)
Dietitian	1.4% (4)
Psychologist	1.4% (4)
Respiratory therapist	1.0% (3)
Dental hygienist	1.0% (3)
Nurse practitioner	1.0% (3)
Pharmacy technician	1.0% (3)
Dental assistant	0.3% (1)
Medical radiation technologist	0.3% (1)

### Measures

#### 12-Item Expectations Regarding Aging Survey (ERA-12; Sarkisian et al., 2005)

The ERA-12 is a shortened (Sarkisian et al., 2005) version of the ERA-38 (Sarkisian et al., 2002) and was used to assess Healthcare professionals’ attitudes toward aging. Participants are asked to indicate how they feel about a given statement using a 4-point Likert scale, ranging from 1 (definitely true) to 4 (definitely false), total scores range from 16 to 48. Higher expectations regarding aging are reflected by higher scores. The scale was found to have good internal reliability, in a sample of primary care clinicians (α = .82) (Davis et al., 2011). The total scale demonstrated an alpha coefficient of .84 in the current study.

#### Ten-Item Perceived Stress Scale (PSS-10; Cohen et al., 1983)

The PSS-10 was used to assess participants’ levels of perceived stress (Cohen et al., 1983). The measure consists of ten items and asks participants to reflect on their feelings over the past month. This is done using a 5-point Likert scale, ranging from 0 (never) to 4 (very often). Total scores fall between 0 and 40. Higher scores on this measure represent higher levels of perceived stress and lower scores represent lower levels of perceived stress. The original scale demonstrates good internal reliability, with Cronbach’s alpha ranging from .84 to .86, as well as good test-retest reliability, with values commonly exceeding .7 (Lee, 2012). In the current study, the scale demonstrated very good internal reliability (α = .92).

#### The Abbreviated Maslach Burnout Inventory (aMBI; McManus et al., 2002; McManus et al., 2000)

The aMBI is a shortened version of the Maslach Burnout Inventory Human Service Scale (MBI-HSS) (McManus et al., 2000; McManus et al., 2002). It is based on a 7-point Likert scale ranging from 0 (never) to 6 (every day). It includes three subscales (depersonalization, emotional exhaustion, and personal accomplishment), with higher scores on depersonalization and emotional exhaustion indicating higher levels of burnout. However, higher personal accomplishment scores indicate lower levels of burnout. All subscales have demonstrated acceptable-to-good internal reliability, with values ranging from .72 to .89 and good concurrent validity compared to the MBI-HSS (Riley et al., 2018; Shaikh et al., 2019). In the current study, Cronbach’s α = .85 for the emotional exhaustion subscale; Cronbach’s α = .72 for the depersonalization subscale; Cronbach’s α = .66 for the personal accomplishment subscale; and Cronbach’s α = .84 for overall burnout.

#### Emotional Dissonance Subscale of the Emotional Labor Scale (Andela et al., 2015)

The emotional dissonance subscale of the emotional labor scale (Andela et al., 2015) consists of four items. Participants are asked to provide a rating from 1 (never) to 5 (always) based on how often their feelings are aligned with the statement provided (Andela et al., 2015). Total scores for emotional dissonance range from 4 to 20. Higher scores are associated with greater emotional dissonance. The subscale has demonstrated good internal reliability within three different samples of healthcare professionals, with alpha coefficients ranging from .81 to .91 (Andela et al., 2015, 2016). In the current study, Cronbach’s α = .79.

#### The WHO-5 Well-Being Index (Topp et al., 2015)

The WHO-5 well-being index provides a subjective measure of a participant’s psychological well-being (Topp et al., 2015). It consists of five items that assess a participant’s feelings over the previous two weeks and is based on a six-point Likert scale. Total scores range from 0 to 25. An example of an item is “I have felt active and vigorous.” The scale has previously demonstrated very good internal reliability (α = .91) (MacIntyre et al., 2020). The reliability was similar for the current study (α = .87).

#### Analyses

To answer the first research question, asking if work experience and profession influences stress, burnout, and expectations regarding aging a few analyses were done. First, Pearson correlations were computed to determine if years of work experience are related to scores on the PSS-10, overall burnout, and ERA-12, years of work experience were evaluated as a continuous variable. Secondly, to understand the role that profession plays in this, a one-way ANOVA was used to determine if professionals significantly differed in their expectations regarding aging. Due to small professional groups, only those with 20 or more participants were included. Lastly, to answer this question, a MANOVA was performed using the physical health, mental health, and cognitive functioning subscales of the expectations regarding the aging scale as dependent variables and profession as the independent variable.

To answer the second question asking about the relationship between stress and ageism, Pearson correlations were also used to examine the relationships between the ERA-12 and overall burnout, as well as the three burnout dimensions. All variables were evaluated as continuous.

**Figure 2.**
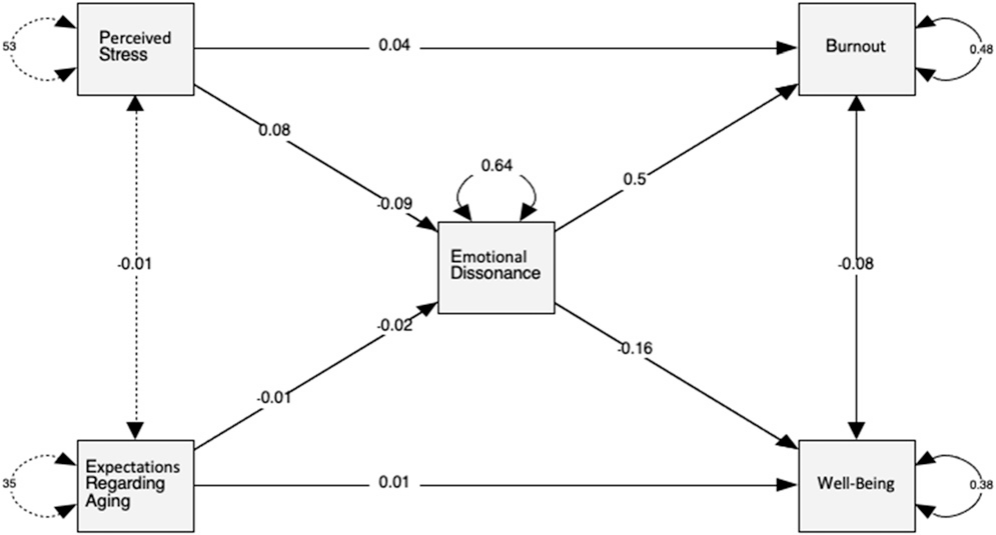
Path analysis of the relationships between mental well-being and expectations regarding aging.

Two answer the two proposed hypotheses—the final analysis of the current study consisted of a path analysis to examine the significance of hypothesized directional relationships between the study variables. Perceived stress (PSS-total) and expectations regarding aging (ERA-total) were used as predictor variables. Emotional dissonance was used as a mediator (ED-total), and both well-being (WHO-total) and overall burnout (BO-total) were the outcome variables. A visual of this model including path coefficients can be found in Figure 2. This path analysis did control for age, gender, and marital status.

### Results

To answer the first research question, results showed that years of work experience are negatively correlated with the PSS-10 (r = −.23, *p* < .001) and overall burnout scores (r = −.32, *p* < .001). That is, less experienced healthcare professionals tend to experience more perceived stress and burnout. There was no significant relationship with scores on the ERA-12 (r = −.04, *p* = .275).

Results of the one-way ANOVA with equal variances were assumed based on the results of Levene’s test [Levene’s F (4, 219) = 1.045*, p* = .385], revealed a significant effect at the univariate level [F (4, 219) = 7.089, *p* < .001]. Post hoc analyses were performed using Tukey’s HSD. Results showed that PTs (M = 37.10, SD = 4.81) have significantly higher or better expectations regarding aging than RNs (M = 33.10, SD = 6.22, *p* = .005), LPNs (M = 31.17, SD = 6.93, *p* < .001), and paramedics (M = 30.46, SD = 5.18, *p* < .001). However, PTs did not differ from SLPs (M = 34.09, SD = 5.37). There were no other significant differences.

As a follow-up analysis, a MANOVA was performed using the physical health, mental health, and cognitive functioning subscales of the expectations regarding the aging scale as dependent variables and profession as the independent variable. Box’s M was non-significant for this analysis [Box’s M = 37.736, F (24, 47,550.556) = 1.512, *p* = .052]. Pillai’s trace was shown to be significant at the multivariate level [V = .200, F (12, 657) = 3.921, *p* < .001]. Equal variances were assumed for physical health [Levene’s F (4, 219) = .281, *p* = .890] and cognitive functioning [Levene’s F (4, 219) = .71, *p* = .585], but not for mental health [Levene’s F (4, 219) = 2.52, *p* = .042]. At the univariate level, the effect of the profession was significant for physical health [F (4, 219) = 5.44, *p* < .001], mental health [F (4, 219) = 6.80, *p* < .001], and cognitive functioning [F (4, 219) = 4.10, *p* = .003]. (Tables 2 and 3)

**Table 2.** Descriptive Statistics of Stress, Burnout, and Expectations Regarding Aging, Emotional Dissonance and Well-Being.

Descriptive Statistics	*N*	Mean	Std. Deviation	Minimum	Maximum
Expectations regarding ageism - total	290	33.401	5.938	16.000	48.000
ERA-total - adjusted to 100	290	59.448	16.496	11.111	100.000
Physical health	290	10.469	2.409	4.000	16.000
Mental health	290	12.460	2.399	4.000	16.000
Cognitive	290	10.472	2.583	4.000	16.000
Perceived stress scale	294	19.925	7.291	3.000	37.000
WHO-5 well-being index	283	11.005	4.817	1.000	23.000
Emotional dissonance subscale of the ELS	287	13.235	3.043	4.000	20.000
aMBI - burnout measure - total	278	15.605	8.476	0.000	35.000
Personal accomplishment	278	13.715	3.385	2.000	18.000
Depersonalization	278	4.501	4.485	0.000	18.000
Emotional exhaustion	278	11.104	5.124	0.000	18.000

**Table 3.** Frequencies of Stress, Burnout, and Expectations Regarding Aging.

Variable	Frequency
	% (*N*)
Perceived stress	
Low (0–13)	22.8% (67)
Moderate (14–26)	59.9% (176)
High (27–40)	17.3% (51)
Perceived stress	
Low (0–9)	9.2% (27)
Moderate (10–19)	35.7% (105)
High (20 or above)	55.1% (162)
Overall burnout	
No-to-low	60.4% (168)
Moderate-to-severe	39.6% (110)
Emotional exhaustion	
No-to-low	36.0% (100)
Moderate-to-severe	64.0% (178)
Depersonalization	
No-to-low	84.2% (234)
Moderate-to-severe	15.8% (44)
Personal accomplishment	
No-to-low	88.1% (245)
Moderate-to-severe	11.9% (33)
Expectations regarding aging	
Low	42.1% (122)
High	57.9 (168)

Tukey’s HSD was used to perform post hoc analyses for all three subscales of expectations regarding aging. Aligned with the aforementioned results, PTs have significantly more positive expectations regarding physical health than registered nurses, licensed practical nurses, and paramedics. The comparison between PTs and SLPs approached significance. PTs and SLPs both demonstrated more positive expectations about mental health than LPNs and paramedics, but not RNs. Finally, only one comparison was significant for expectations of cognitive functioning, such that physiotherapists held higher expectations than paramedics. Descriptive statistics can be found in Table 4 and a breakdown of the post hoc analyses can be found in Table 5.

**Table 4.** Descriptive Statistics for ERA Subscales by Profession.

Profession	Physical Health		Mental Health		Cognitive Function	
	M	SD	M	SD	M	SD
RN	10.39	2.45	12.19	2.42	10.53	2.60
LPN	9.79	2.32	11.00	3.26	10.38	2.57
PT	12.01	2.34	13.39	1.98	11.69	2.55
SLP	10.43	2.38	13.45	2.01	10.21	2.84
Paramedic	9.72	2.43	11.44	2.14	9.31	2.34

**Table 5.** Post Hoc Analyses for ERA Subscales.

Profession	Profession	Physical Health	Mental Health	Cognitive Function
		Mean Difference	Mean Difference	Mean Difference
RN	LPN	.59	1.19	.16
	PT	−1.62**	−1.20	−1.16
	SLP	−.05	−1.26	.32
	Paramedic	.67	.75	1.22
LPN	PT	−2.22**	−2.39**	−1.32
	SLP	−.64	−2.45**	.17
	Paramedic	.07	−.44	1.07
PT	SLP	1.57	−.06	1.49
	Paramedic	2.29***	1.95**	2.39***
SLP	Paramedic	−.72	2.01**	−.90

*Note.* ***p < .001, **p < .01, and *p < .05.

To answer the second research question, results indicated that lower ERA-12 scores were significantly correlated with greater overall burnout, emotional exhaustion, and depersonalization. Furthermore, lower scores on the ERA-12 were significantly related to greater reductions in personal accomplishment (Table 6). Similarly, correlations determined the relationships between stress and expectations regarding aging, and overall burnout and well-being. Correlations were computed as a preliminary analysis for all mental well-being variables (i.e., perceived stress, burnout, emotional dissonance, and well-being; Table 7). All correlations were found to be significant. Expectations regarding aging demonstrated weak-moderate relationships with each of the mental well-being variables, with Pearson correlations ranging from −.23 to −.28. Notably, expectations regarding aging were significantly related to perceived stress, such that poorer aging expectations were related to higher perceived stress (r = −.28, *p* < .001). In addition, overall burnout, perceived stress, and emotional dissonance were all strongly, and positively related to one another (between r = .58 and .68). However, well-being was strongly, but negatively correlated to all three of these variables (between .56 and .78. Thus, higher burnout is related to poorer well-being.

**Table 6.** Relationships Between Expectations Regarding Aging and Burnout.

Variable		1	2	3	4	5
1. Expectations regarding aging	Pearson’s r					
2. Overall burnout	Pearson’s r	−0.282*				
3. Emotional exhaustion	Pearson’s r	−0.242*	0.898*			
4. Depersonalization	Pearson’s r	−0.256*	0.864*	0.554*		
5. Personal accomplishment	Pearson’s r	0.228*	−0.474*	−0.365*	−0.479*	

*Note.* *p < .001.

**Table 7.** Pearson Correlations for Mental Well-Being and Expectations Regarding Aging.

Variable		1	2	3	4	5
1. Overall burnout	Pearson’s r					
2. Emotion dissonance	Pearson’s r	0.675*				
3. Expectations regarding aging	Pearson’s r	−0.282*	−0.226*			
4. Perceived stress	Pearson’s r	0.579*	0.591*	−0.275*		
5. Well-being	Pearson’s r	−0.594*	−0.563*	0.275*	−0.776*	

*Note.* *p < .001.

Lastly, to test the two hypotheses, results of the path analysis show that stress significantly predicts emotional dissonance (β = 0.08, *p* < .001), and in turn, emotional dissonance predicts overall burnout (β = .50, *p* < .001). As previously mentioned, stress and burnout have a strong, positive correlation (r = .58); however, when emotional dissonance is included as a mediator, this relationship is significantly reduced (β = .04, *p* < .001). Likewise, emotional dissonance significantly predicts levels of well-being (β = −.16, *p* < .001), and the relationship between stress and well-being is significantly reduced within the path analysis. That is, the initial correlation coefficient of −.78 decreases when emotional dissonance is accounted for in the relationship (β = −.09, *p* < .001). Thus, emotional dissonance partially mediates the relationship stress has with both overall burnout and well-being.

The same path analysis was also used to determine whether emotional dissonance mediated the relationships that expectations regarding aging share with overall burnout and well-being. As mentioned, emotional dissonance predicts both burnout and well-being; however, expectations regarding aging did not significantly predict emotional dissonance (β = −.01, *p* = .18), despite its significant correlation (r = −.23). Thus, expectations regarding aging do not predict emotional dissonance when stress is also accounted for as a predictor. As a result, emotional dissonance does mediate the relationship with overall burnout or well-being. A full breakdown of the direct, indirect, and total effects can be found in Tables 8–10.

**Table 8.** Direct Effects.

	Estimate	Std. Error	z-Value	*p*	95% Confidence Interval	
					Lower	Upper
PSS-total → BO-total	0.035	0.007	4.880	<.001	0.017	0.052
ERA-total → BO-total	−0.016	0.007	−2.230	.026	−0.034	2.083 × 10^−5^
PSS-total → WHO-total	−0.092	0.006	−14.524	<.001	−0.105	−0.077
ERA-total → WHO-total	0.009	0.006	1.436	.151	−0.004	0.023

*Note.* Delta method standard errors, bias-corrected percentile bootstrap confidence intervals, and ML estimator.

**Table 9.** Indirect Effects.

	Estimate	Std. Error	z-Value	*p*	95% Confidence Interval	
					Lower	Upper
PSS-total → ED-total → BO-total	0.039	0.005	7.422	<.001	0.030	0.049
ERA-total → ED-total → BO-total	−0.006	0.004	−1.330	.183	−0.014	0.002
PSS-total → ED-total → WHO-total	−0.012	0.004	−3.299	<.001	−0.021	−0.004
ERA-total → ED-total → WHO-total	0.002	0.001	1.251	.211	−3.650 × 10^−4^	0.006

*Note.* Delta method standard errors, bias-corrected percentile bootstrap confidence intervals, and ML estimator.

**Table 10.** Total Effects.

	Estimate	Std. Error	z-Value	*p*	95% Confidence Interval	
					Lower	Upper
PSS-total → BO-total	0.074	0.007	10.836	<.001	0.059	0.089
ERA-total → BO-total	−0.022	0.008	−2.609	.009	−0.041	−0.003
PSS-total → WHO-total	−0.104	0.005	−19.515	<.001	−0.114	−0.094
ERA-total → WHO-total	0.011	0.007	1.678	.093	−0.002	0.025

*Note.* Delta method standard errors, bias-corrected percentile bootstrap confidence intervals, and ML estimator.

### Discussion

This study sought to examine the relationships between ageism, stress, and burnout in a group of healthcare providers while considering a novel variable: emotional dissonance. Previous studies have established relationships between ageism and burnout (e.g., Hakim, 2021). Additionally, emotional dissonance has previously been found to mediate the relationship between stress and burnout (e.g., Kwak et al., 2018). The present study examined emotional dissonance as a mediator between an overall perception of stress and burnout, in addition to ageism.

As noted above, some have found that there is a relationship between burnout and ageism in nurses (e.g., Iecovich & Avivi, 2017). This relationship has yet to be investigated in a sample of diverse healthcare professionals. In this study, higher overall burnout, emotional exhaustion, and depersonalization were associated with poorer expectations regarding aging. Those with poorer expectations experienced lower levels of personal accomplishment. Like Hakim (2021) who found that ageist attitudes are associated with higher levels of burnout. In the current study, the relationships between ageism and emotional exhaustion and overall burnout are noticeably weaker. This could be because Hakim (2021) used Kogans Attitudes Toward Old People Scale (Kogan, 1961), whereas the current study used the 12-Item ERA (Sarkisian et al., 2005).

Beyond burnout, the current study investigated if expectations regarding aging share similar relationships with stress and well-being. Results revealed that poor expectations of aging were related to higher levels of stress and poorer well-being. The strength of these relationships was similar in strength to that of burnout. This could be because those who have poorer attitudes toward aging may experience higher levels of emotional dissonance when working with older adults. The elevation of emotional dissonance may then reduce one’s well-being and increase the sense of burnout (Iecovich & Avivi, 2017). The current study found that higher emotional dissonance was strongly related to poorer well-being and higher burnout. It was also related to higher levels of perceived stress. These relationships have also been established in previous literature (e.g., Andela et al., 2016); however, these studies focused on work-related stress, rather than a general perception of stress. Importantly, poorer expectations regarding aging in the current study, indicating higher ageism, were related to higher levels of emotional dissonance experienced by healthcare professionals.

However, the results of the path analysis were not aligned with the research hypotheses. When expectations regarding aging and stress were both included as predictor variables, poor aging expectations no longer predicted higher emotional dissonance. Thus, emotional dissonance did not mediate the relationship between ageism and burnout as hypothesized by Iecovich and Avivi (2017). This could be because the current study did not target healthcare professionals working specifically with older populations. However, given the aging population in the Atlantic provinces (Government of Canada, 2022a), and the higher likelihood of older adults accessing healthcare services (Government of Canada, 2022b; Kwok et al., 2016), it is likely that the majority of our sample worked with older adults.

Expectations regarding aging did not vary based on the years of experience of healthcare professionals. Previous studies suggested that less experienced professionals tend to hold more negative attitudes toward aging (Leung et al., 2011; Schroyen et al., 2016). However, Davis et al. (2011) found that using the same expectations regarding aging scale, older clinicians, likely with more experience, demonstrated poorer expectations. This should be interpreted with caution as the literature is mixed regarding the influence of age and work experience on ageism.

When examining the difference between professions, PTs demonstrated substantially more positive expectations regarding aging than RNs, LPNs, and paramedics. In follow-up analyses, PTs differed only from LPNs and paramedics on mental health expectations, and paramedics on cognitive function. Most importantly, it was determined that physical therapists only significantly differed from all three professions based on physical health. Additionally, the difference with SLPs neared, but did not reach, significance. Physiotherapists are mainly concerned with the physical health of their patients, and aim to restore proper function, (Bruschini et al., 2018). They are also dealing with individuals in a restorative/rehabilitation manner and are thus not seeing older adults in urgent acute situations, unlike other healthcare professionals, notably, paramedics. Aspects of their work also require an understanding of psychological concepts such as cognitive disabilities (Bruschini et al., 2018). The more positive attitudes toward the physical health of older adults on behalf of PTs may demonstrate the nature of their work. Other explanations focus on differences in knowledge, where nurses were found to be the least knowledgeable about and had little education on older adults (Wells et al., 2004). They were also more likely to relate the process of aging to frailty and boredom, which may coincide with the poorer expectations of physical health in both LPNs and RNs, and mental health in LPNs.

The results of this study found that healthcare professionals in the Atlantic provinces are experiencing high rates of stress, burnout, and poor well-being and there is a relationship with ageism. The existence of this relationship is troubling. It is important to be aware that the attitudes of healthcare professionals may have negative effects on their mental well-being, and vice versa. Appropriate interventions in future research should focus on uncovering ways to address these attitudes so they do not become problematic to employees or those they provide care to. Improved education about older adults may be a key factor in improving expectations regarding aging among healthcare professionals (Wells et al., 2004). Kagan and Melendez-Torres (2015) suggest that successful interventions should address false expectations and information about the aging process. Also, given the possibly uneven exposure to older adults in an unhealthy state, ageism may be further combatted by allowing opportunities for professionals or students to gain experience healthy with older adults (Leung et al., 2011). Future studies and implementation of such interventions are required to determine their effectiveness within the healthcare setting.

### Conclusion

The study’s cross-sectional design poses a limitation, as it hinders the confirmation of directional relationships without longitudinal data, despite employing path analysis to infer hypothetical causal links. The limited sample size, attributed to the challenges of studying a relatively small population in Atlantic Canada, further constrains the analysis. A larger sample size could have provided more power and potentially yielded different results. It is important to acknowledge the practical realities of researching a region with fewer available participants, especially healthcare professionals. Also, nine in ten of the participants were women, a gender distribution that tends to remain true in many areas of the Canadian healthcare system. Approximately 94% of speech-language pathologists, 92% of registered nurses/registered psychiatric nurses, 91% of licensed practical nurses, and 75% of physiotherapists in the country are women (Statistics Canada, 2022). These four professionals were among the most frequently reported in the current sample, explaining the higher sampling of women. These differences prevented the current study from evaluating gender differences. Importantly, this may have affected the prevalence rates within the current sample. Women may report higher levels of emotional exhaustion, but less depersonalization and reduced personal accomplishment than males (e.g., Cañadas-De la Fuente et al., 2015). This may explain the relatively low rates of depersonalization and diminished personal accomplishment in the current sample. Future studies should study a more diverse gender sample of healthcare professionals.

This study did include diverse professions in healthcare, which allowed previously studied relationships among a small subset of professionals (e.g., nurses) to be understood in a larger healthcare context. However, the work setting or client population was not controlled for, and some professions where unable to be compared due to the sample size. Thus, it was not possible to determine if differences in the relationships may exist based on such factors. Future studies should consider including comparisons based on these variables. This may be useful to enhance understanding of the relationship between ageism and burnout. Also, participation among included professions varied greatly, resulting in an unequal representation.

The current study was unable to provide support for the hypothesis that emotional dissonance mediates the relationship between ageism and burnout. Consequently, further research is required to determine what, if any, variables explain the relationship between ageism and burnout. A suggested approach may be to explore the possibility that increasing levels of burnout may lead to the development of ageist attitudes. Specifically, it seems reasonable that depersonalization, which can affect a professional’s attitudes toward their clients and patients (Maslach & Jackson, 1981), could lead to the development of such attitudes.

Moreover, although Atlantic Canadian healthcare professionals displayed relatively neutral to positive expectations regarding aging, there are significant differences among certain professions. Importantly, this study was able to expand upon previous literature by finding that a professional’s attitudes toward aging tend to become poorer as they experience more burnout, and vice versa. To our knowledge, this is the first time this relationship has been confirmed within a diverse sample of healthcare professionals. However, the study was unable to conclude that this relationship can be explained through emotional dissonance.

This study was able to demonstrate that healthcare professionals in the Atlantic provinces are experiencing high rates of stress, burnout, and poor well-being. With these issues, comes a host of potentially detrimental issues for healthcare employers and their older clients and patients. Therefore, the study draws attention to the immediate need to improve the well-being of healthcare professionals to decrease ageism in the healthcare systems.
